# Preventing railway suicides through level crossing removal: a multiple-arm pre-post study design in Victoria, Australia

**DOI:** 10.1007/s00127-022-02340-9

**Published:** 2022-07-21

**Authors:** Angela Clapperton, Jeremy Dwyer, Matthew J. Spittal, Leo Roberts, Jane Pirkis

**Affiliations:** 1 Melbourne School of Population and Global Health, The University of Melbourne, Carlton, VIC Australia; 2 Coroners Prevention Unit, Coroners Court of Victoria, Melbourne, VIC Australia

**Keywords:** Suicide, Railway, Case–control study, Epidemiology, Intervention studies

## Abstract

**Purpose:**

Rail level crossing removals to improve transport performance across metropolitan Melbourne (state of Victoria) resulted in new rail fencing and grade-separation of tracks from the surrounding environment at several sites. These design changes restricted pedestrian access to the rail tracks, which is a countermeasure known to prevent railway suicide in other settings. We examined whether any such suicide prevention effect followed the removals.

**Methods:**

We used a multiple-arm pre-post design to test whether a decrease in monthly frequency of railway suicides occurred at level crossing removal sites (intervention sites), compared to randomly matched sites where level crossings had not yet been removed (control sites). We used data available in the Victorian Suicide Register covering the period 1st January 2008 to 30th June 2021.

**Results:**

The mean monthly number of railway suicides decreased by 68% within a 500 m radius of intervention sites (RR: 0.32; CI 95% 0.11–0.74) and by 61% within a 1000 m radius of intervention sites (RR: 0.39; CI 95% 0.21–0.68). There was no evidence that the mean monthly number of railway suicides changed at the control sites, either within a 500 m radius (RR: 0.88; CI 95% 0.47–1.56) or a 1000 m radius (RR: 0.82; CI 95% 0.52–1.26).

**Conclusion:**

The reduction in railway suicides at locations where level crossings were removed, demonstrates the suicide prevention benefits that can be derived from a major infrastructure project even if not initially intended. Planning for major infrastructure projects should include consideration of these benefits, with designs incorporating features to maximise suicide prevention impact.

## Introduction

Railway suicide accounts for an estimated 1–12% of all suicides internationally [1]. While all suicides are concerning and warrant prevention efforts, railway suicides are of particular concern because they occur at public sites and are therefore often witnessed by people who are working at or using the rail network, or those or who are simply near to the railway when an incident occurs. Train drivers can experience severe psychological consequences (e.g., acute stress reactions and PTSD) after being involved in a railway suicide [2–4]. Preventing suicide attempts in the rail environment is also a high priority as attempts commonly result in serious injury [5], have a high case-fatality rate [6], and have substantial financial implications including through having major effects on the normal operation of the railway system [7, 8].

Restricting access to means is one intervention that has been shown to be effective in preventing suicides at public places in general [9–11], and also in the rail environment [7, 12–15]. Restricting access to means is thought to be effective because it interrupts the suicidal process, giving the individual time to rethink their actions and/or allowing others to intervene [16]. An example of an intervention to reduce access to means in the rail environment is the installation of platform screen doors which open only when a train stops at the station, therefore preventing individuals from accessing the track. Research in Hong Kong [12, 13] Seoul [15], Tokyo [14] and Shanghai [7] has consistently identified large reductions in railway suicide after the installation of platform screen doors (range 60–91%).

Australia has high railway suicide rates when compared with other countries [17] and the state of Victoria accounts for 46% of Australian suicides resulting from jumping or lying before a moving object [18] (mostly trains) [19]. This is despite the fact that Victoria accounts for just 26% of the population and 22% of Australian suicides overall [18]. It is estimated that most of the railway network is unfenced in Victoria, and consequently, close to 70% of railway suicides take place on open tracks, including 11% at level crossings [20].

Level crossings occur where the road (and often footpaths) intersect with the rail track. Removing level crossings, either by lowering railway tracks under roads or by building new rail bridges or sections of track over roads, might act as one way of restricting access to means of suicides at these level crossing sites. We were able to test the effectiveness of this as a strategy by capitalising on the Level Crossing Removal Project which was established by the Victorian Government in 2015 to oversee the removal of 85 level crossings across metropolitan Melbourne by 2025. The crossings are being removed with the aim of reducing traffic congestion, improving travel time reliability, increasing capacity to run more trains on the network and improving safety [21]. The last aim of improving safety may have specific benefits for suicide prevention because it involves removing the danger of trains sharing a crossing with vehicles and pedestrians who may be on the track. We used real-time suicide data to determine whether the incidence of railway suicides changed at sites where level crossings were removed.

## Methods

### Study design

We used a multiple-arm pre-post design to test whether the removal of level crossings was associated with a decrease in railway suicides within a 500 m radius and 1000 m radius of the level crossing removal sites. We identified sites where level crossings had been removed (intervention sites) and randomly matched these to sites where level crossings had not yet been removed (control sites). For each intervention and control site pair, we compared the number of railway suicides per month before and after the period that the crossing was removed at the intervention site, by calculating rate ratios. The observation period covered by the study was 1st January 2008 to 30th June 2021.

### Data sources

We obtained data for this study from two main sources: the Victorian Level Crossing Removal Project official website (https://levelcrossings.vic.gov.au/) and the Victorian Suicide Register (VSR).

We searched the Victorian Level Crossing Removal Project official website for all level crossing removals that had occurred prior to 31st December 2020. We identified 41 sites where the level crossings had been removed and we used information on the website to determine the start and finish dates for each removal. If these dates were not clearly provided, we gathered this information through internet searches (e.g., news media articles). We then randomly matched these 41 intervention sites to 41 control sites where the level crossings were due to be removed but had not yet been removed (as of 30th June 2021).

We used the VSR to obtain data on railway suicides occurring near to the intervention and control sites. The VSR was established by the Coroners Court of Victoria (‘the Court’). In Victoria all deaths from suspected non-natural causes are required to be reported to the Court for investigation. Trained coders review what is known about the circumstances of each death upon initial report, to identify suspected suicides; these are added to the VSR [22]. Factors considered in the review process include mechanism of death, evidence of suicidal intent, stressors the deceased was experiencing, and the likelihood of alternate scenarios to explain how and why the death occurred. The deaths included as suicides in the VSR are regularly reviewed as coroners’ investigations progress and more is known about them; deaths may be removed if deemed not to be suicides, or may be added if new evidence suggests they were wrongly excluded on initial review [23]. The VSR contains basic coded information for every suspected suicide reported to the Court since 1st January 2000, including data of fatal incident; deceased sex, age, country of birth and location of usual residence; location (including latitude and longitude) of the fatal incident; and suicide method. We used the longitude and latitude data to identify all railway suicides occurring within a 500 m radius and a 1000 m radius of the intervention and control sites from 1st January 2008 to 30th June 2021. Railway suicides that occurred during the period of the removal of the crossings were excluded from analysis, so that the pre-period used for analysis included full months prior to any level crossing removal work beginning and the post-period included full months covering the period when the work had concluded.

### Analysis

We calculated rate ratios for the 41 intervention sites at which the level crossing had been removed and the 41 control sites where the level crossing was yet to removed. The rate ratio (RR) compared railway suicide in the pre- and post-intervention periods for control and intervention sites. We did this for railway suicides within a 500 m radius of the level crossing site and 1000 m radius of the level crossing site. To test whether the rate ratio for the intervention sites was different from the rate ratio for the control sites we used a Poisson regression model which included variables for control vs. intervention site, pre- vs. post-period and the interaction between these two. We also included the corresponding duration of these time periods as an offset term.

### Ethics

The study was reviewed and approved by the University of Melbourne’s Human Research Ethics Committee (Reference Number: 2021-22015-21133–3).

## Results

Between 1st January 2008 and 30th June 2021, we identified 139 railway suicides that occurred within a 500 m radius of an intervention or control level crossing site, and 266 railway suicides that occurred within a 1000 m radius of an intervention or control level crossing site (Table 1). During the period prior to any level crossing removals, there was no evidence of a difference in the number of railway suicides occurring within a 500 m or 1000 m radius of intervention sites when compared to control sites (500 m: RR = 1.05, 95% CI 0.72 to 1.53, *p* = 0.78; 1000 m: RR = 1.05, 95% CI 0.80 to 1.38, *p* = 0.68).

**Table 1:** Number of railway suicides occurring within a 500 m and 1000 m radius of intervention and control sites and rate ratios (RR) comparing the pre- and post-intervention periods, Victoria, January 2008 to June 2021

	Pre-period (Total 4724 months)		Post-period (Total 1472 months)			
	Number of railway suicides	Railway suicide rate (per month)	Number of railway suicides	Railway suicide rate (per month)	RR (95% CI)	
500 m radius of site						
• Intervention sites	60	0.013	6	0.004	0.32 (0.11, 0.74)	*p* = 0.003*
• Control sites	57	0.012	16	0.011	0.88 (0.47, 1.56)	*p* = 0.725
Ratio comparing intervention to control sites (500 m radius)					0.36 (0.13, 0.97)	*p* = 0.044**
1000 m radius of site						
• Intervention sites	115	0.024	14	0.010	0.39 (0.21, 0.68)	*p* = 0.001*
• Control sites	109	0.023	28	0.020	0.82 (0.52, 1.26)	*p* = 0.360
Ratio comparing intervention to control sites (1000 m radius)					0.47 (0.24, 0.95)	*p* = 0.035**

*Significant decrease

**Significant difference between the two incident rate ratios

We found evidence that the number of railway suicides within 500 m radius of a level crossing decreased by 68% after removal of the level crossing (RR: 0.32; CI 95% 0.11–0.74). In contrast, we found no evidence that the number of railway suicides changed at the control sites (RR: 0.88; CI 95% 0.47–1.56). Considering these two findings together, the number of railway suicides within a 500 m radius of sites was 64% lower in the post-intervention period at the sites where level crossings were removed than at the control sites (RR: 0.36; CI 95% 0.13–0.97, *p* = 0.044).

Results were very similar when we examined railway suicides within a 1000 m radius of a level crossing; railway suicides decreased by 61% after removal of the level crossing (RR: 0.39; CI 95% 0.21–0.68) and there was no evidence that the number of railway suicides changed at the control sites (RR: 0.82; CI 95% 0.52–1.26). In this case, the number of railway suicides within a 1000 m radius of sites, was 53% lower in the post-intervention period at the level crossing removal sites than at the control sites (RR: 0.47; CI 95% 0.24–0.95, *p* = 0.035).

## Discussion

We examined the effectiveness of removing level crossings as a railway suicide prevention measure. Through analysis of real-time suicide surveillance data in Victoria, we found good evidence of a reduction in railway suicides within a 500 m radius and 1000 m radius of level crossing removal sites (68% and 61% decrease in the number of suicides, respectively). In contrast, there was no evidence of a change in the number of railway suicides at the control sites, either within a 500 m radius or within a 1000 m radius of the sites.

At many of the intervention sites in our study, the level crossing removals resulted in the railway track becoming completely inaccessible to pedestrians and car drivers, because long sections of railway track were grade-separated from the surrounding environment (either raised above ground or lowered underground). Further to this, authorities typically installed safety fences while the work was taking place and at many sites some fencing remained in place following completion. For these reasons it is perhaps unsurprising that we found railways suicides reduced within 500 m radius of the level crossing sites: the fencing and grade separation acted to reduce access to the track. Reducing access to means is a suicide prevention intervention with a substantial amount of supportive evidence. In rail settings, it has already been shown effective through the installation of screen doors on train platforms [12–14].

However, the fact that we observed a reduction in railway suicides within a 1000 m radius of the site (i.e., effectively two kilometres of track) suggests the effect may not be confined to the immediate location where level crossings were removed. Research with people with a lived experience of suicide attempt/s on the railway has identified the perceived lethality of these attempts and the ease of access to tracks as key reasons for using the railway as the location and the method of a suicide attempt [24]. Perhaps, by reducing access at some sites along the network, this may have made surrounding track lengths also appear safer, and could have deterred individuals from attempting suicide at other nearby sites on the railway network.

Although we were unable to examine the potential displacement effects of individuals moving to another location on the railway, or substitution effects of individuals using other suicide methods, existing evidence suggests that when access to means is reduced, there is relatively minimal displacement of suicides to a different location where the same method is typically used [25, 26]. Also, individuals are unlikely to substitute or change suicide method if their “preferred” method is unavailable [27], or if they do, they often use less lethal methods [28, 29] which would presumably be especially true given the lethality of railway suicide attempts. In our study we found no evidence of an increase in suicides at the control sites where the level crossings were yet to be removed. Many of the control sites were near to the level crossing removal sites, which could potentially provide some reassurance that people are at least not moving to other nearby level crossing sites on the rail network to attempt suicide.

### Strengths and limitations

Our study is to our knowledge the first to investigate the impact of level crossing removals on railway suicide. We used real-time surveillance data which allowed us to measure this impact even as the Level Crossing Removal Project was still underway. Another strength of the study is that we used accurate, manually assigned, geocoded suicide location data rather than relying on data based on auto-geocoding processes which are known to be inaccurate, especially for non-residential addresses which are often erroneously geocoded to centralised fall-back locations [30].

This study has provided useful information regarding the suicide prevention potential for level crossing removals and other similar infrastructure projects which may restrict access to rail tracks. However, it has some limitations. Data regarding the exact dates associated with the removal of level crossings were not always readily available so some assumptions had to be made using data gathered from other sources such as newspaper articles. However, we are confident that our pre period included only full calendar months prior to the removal work beginning and that our post period included only full calendar months following the completion of the removal work.

We were unable to demonstrate whether the current level crossing removals have had an impact on overall railway suicide numbers in Victoria as we completed this study when fewer than half of the planned level crossing removals had taken place. Future research should revisit this analysis as more level crossings are removed as there is the possibility that progressively making the railway network safer through additional level crossing removals may eventually influence railway suicide numbers overall, which, along with overall suicide rates, have remaining fairly stable in Victoria over recent years [18].

Multiple studies regarding railway suicide have shown that using half-height platform screen doors (which do not extend to the ceiling of the platform and therefore do not fully restrict access to the railway track) is a less effective suicide prevention measure than using screens that extend to the ceiling [7, 14, 15]. The superior results of interventions that completely, rather than partially, restrict access to means, has also been demonstrated in other public places where other methods of suicide, such as jumping from a height, are common [31]. We did not examine whether there were differences in the suicide prevention effect of removing level crossings depending on whether the track has been made completely inaccessible (i.e., through raising the track above the ground or lowering it underground, or through the inclusion of fencing to prevent access to the track) but this should be a focus of future research.

### Implications and conclusion

Major transport infrastructure initiatives are very expensive; in 2018 Melbourne’s Level Crossing Removal Program carried a total estimated cost of AUD$14.8 billion [32]. While suicide prevention may not be an explicit goal of such initiatives, this study demonstrates how a substantial reduction in suicides on the transport infrastructure may result. For example, in the year before any level crossings were removed, 16 railway suicides occurred within 500 m of a level crossing across the Victorian network. If we assume all crossings were removed at once, and we expect a 68% reduction as we saw at intervention sites, we could expect 55 lives to be saved over the following five years. Although in Australia level crossings are most common across the Victorian rail network, our findings suggest consideration should be given to removing additional level crossings across Australia, especially at sites where suicides have occurred. In addition, when considering and evaluating future plans for transport infrastructure projects, we would advocate for suicide prevention potential and lives saved to be included in the project calculus. Furthermore, for approved transport infrastructure projects moving into the design phase, we would advocate for the design to be informed by a consideration of what features might achieve the biggest suicide prevention impact.

## References

[1] Krysinska K, de Leo D (2008). Suicide on railway networks: epidemiology, risk factors and prevention. Aust N Z J Psychiatry.

[2] Giupponi G (2019). Posttraumatic stress reactions of underground drivers after suicides by jumping to arriving trains; feasibility of an early stepped care outpatient intervention. J Trauma Dissociation.

[3] Farmer R (1992). Railway suicide: the psychological effects on drivers. Psychol Med.

[4] Limosin F (2006). A prospective study of the psychological effects of “person under train” incidents on drivers. J Psychiatr Res.

[5] Virdee J (2018). Mind the gap: 11 years of train-related injuries at the Royal London Hospital Major Trauma Centre. Ann R Coll Surg Engl.

[6] Elnour A, Harrison J (2007). Lethality of suicide methods. Inj Prev.

[7] Xing Y, Lu J, Chen S (2019). Evaluating the effectiveness of platform screen doors for preventing metro suicides in China. J Affect Disord.

[8] Mehnert A (2012). Course and predictors of posttraumatic stress among male train drivers after the experience of 'person under the train' incidents. J Psychosom Res.

[9] Mann JJ (2005). Suicide prevention strategies: a systematic review. J Am Med Assoc.

[10] Pirkis J (2015). Interventions to reduce suicides at suicide hotspots: a systematic review and meta-analysis. Lancet Psychiatry.

[11] Pirkis J (2013). The effectiveness of structural interventions at suicide hotspots: a meta-analysis. Int J Epidemiol.

[12] Law CK (2009). Evaluating the effectiveness of barrier installation for preventing railway suicides in Hong Kong. J Affect Disord.

[13] Law CK, Yip PS (2011). An economic evaluation of setting up physical barriers in railway stations for preventing railway injury: evidence from Hong Kong. J Epidemiol Community Health.

[14] Ueda M, Sawada Y, Matsubayashi T (2015). The effectiveness of installing physical barriers for preventing railway suicides and accidents: evidence from Japan. J Affect Disord.

[15] Chung YW (2016). The effectiveness of platform screen doors for the prevention of subway suicides in South Korea. J Affect Disord.

[16] Azrael D, Miller M, O'Connor RC, Pirkis J (2016). Reducing Suicide Without Affecting Underlying Mental Health. The International Handbook of Suicide Prevention.

[17] European Union Agency for Railways, Railway safety performance in the European Union, E.U.A.f. Railways, Editor. 2020.

[18] ABS, Causes of Death. Catalogue No. 3303.0. 2020, Australian Bureau of Statistics: Canberra, Australia.

[19] Routley, V., et al., Suicide and Natural Deaths in Road Traffic: Review. 2003, Monash University Accident Research Centre: Melbourne, Australia.

[20] Too LS (2015). An investigation of neighborhood-level social, economic and physical factors for railway suicide in Victoria. Australia J Affect Disord.

[21] State Government of Victoria Australia. Project overview - Victoria’s Big Build. 2022 05/07/2022]; Available from: https://bigbuild.vic.gov.au/projects/level-crossing-removal-project/about/project-overview.

[22] Dwyer J (2021). COVID-19 as a context in suicide: early insights from Victoria, Australia. Aust N Z J Public Health.

[23] Sutherland G (2018). Implementation and evaluation of the Victorian Suicide Register. Aust N Z J Public Health.

[24] Marzano L (2019). Factors deterring and prompting the decision to attempt suicide on the railway networks: findings from 353 online surveys and 34 semi-structured interviews. Br J Psychiatry.

[25] Berman AL, Athey A, Nestadt P (2022). Effectiveness of restricting access to a suicide jump site: a test of the method substitution hypothesis. Inj Prev.

[26] Law CK, Sveticic J, De Leo D (2014) Restricting access to a suicide hotspot does not shift the problem to another location. An experiment of two river bridges in Brisbane, Australia. Aust N Z J Public Health 38(2):134–8. 10.1111/1753-6405.12157 24690051

[27] Daigle MS (2005) Suicide prevention through means restriction: assessing the risk of substitution. A critical review and synthesis. Accid Anal Prev 37(4):625–32 10.1016/j.aap.2005.03.004 15949453

[28] Yip P (2012). Means restriction for suicide prevention. Lancet.

[29] de Silva VA (2012). From pesticides to medicinal drugs: time series analyses of methods of self-harm in Sri Lanka. Bull World Health Organ.

[30] Torok M (2021). Spatial errors in automated geocoding of incident locations in Australian suicide mortality data. Epidemiology.

[31] Hemmer A, Meier P, Reisch T (2017). Comparing different suicide prevention measures at bridges and buildings: lessons we have learned from a National Survey in Switzerland. PLoS ONE.

[32] Victorian Auditor-General’s Office, Follow up of Managing the Level Crossing Removal Program, in Victorian Government Printer. 2020: East Melbourne

